# Effect of Nd: YAG laser-fluoride application sequence on enamel demineralization: a micro-CT in vitro study

**DOI:** 10.1007/s10103-026-05080-3

**Published:** 2026-10-27

**Authors:** Angelinne Ribeiro Angelo Gomes, Richard John Heck, Anderson Stevens Leonidas Gomes, Pamella Recco Alvares, Luiz Gustavo de Sousa Duda Júnior, Shamara Pinto Ferreira da Cruz, Ana Marly Araújo Maia, Luiz Alcino Monteiro Gueiros

**Affiliations:** 1 Department of Clinical and Preventive Dentistry, Universidade Federal de Pernambuco, Recife, Brazil; 2 Department of Land Resource Science, University of Guelph, Guelph, Canada; 3 Department of Physics, Universidade Federal de Pernambuco, Recife, Brazil; 4 Department of Oral and Maxillofacial Pathology, Universidade de Pernambuco, Recife, Brazil; 5 Department of Stomatology, Universidade de São Paulo, São Paulo, Brazil; 6 Faculdade de Odontologia do Recife, Recife, Brazil; 7 Department of Dentistry, Universidade Estadual da Paraíba, Campina Grande, Brazil

**Keywords:** Dental caries, Fluorides, topical, Lasers, solid-state, X-ray microtomography, Dental enamel, Cattle

## Abstract

To evaluate, in an in vitro model, whether pulsed Nd: YAG laser irradiation combined with 5% sodium fluoride varnish inhibits bovine enamel demineralization more effectively than fluoride alone, and whether the order of application and the laser power influence the outcome. One hundred and ten bovine enamel specimens were randomly allocated to five groups: Control (C), Fluoride (F), Laser (L), Fluoride-then-Laser (FL), and Laser-then-Fluoride (LF). Irradiated groups received a pulsed Nd: YAG laser at 10 Hz and 300 µs at average powers of 0.5, 0.75, and 1.0 W, with 10 specimens per power subgroup. After treatment, specimens underwent a 48-hour chemical demineralization challenge. Surface loss, measured as lesion step-height in micrometres, and the difference in relative radiodensity between treated and protected enamel were quantified by micro-computed tomography. Data were compared by one-way ANOVA with Tukey’s post hoc test, α = 0.05. The control group showed the greatest surface loss, 89.8 ± 24.6 μm, and the largest radiodensity difference, 1756 ± 746 grayscale units. All treatments except FL0.5 significantly reduced surface loss relative to control. The Laser-then-Fluoride sequence produced the lowest surface loss at every power, from 11.2 to 15.1 μm, but these values did not differ significantly from fluoride alone, which yielded 33.8 ± 23.9 μm. Surface loss did not differ significantly among the three laser powers. The lowest radiodensity differences were recorded in LF1 and F, at 317 ± 401 and 376 ± 292 grayscale units, which did not differ significantly from each other. Applying the laser before fluoride varnish outperformed the reverse sequence, whereas laser power did not influence the outcome. Under the conditions of this in vitro model, the anti-demineralization effect of the best laser-assisted protocol was comparable to that of fluoride varnish alone. Whether laser pretreatment confers a measurable advantage in clinical conditions remains to be determined.

## Introduction

The control of dental caries remains strongly dependent on fluoride-based strategies, which promote enamel remineralization and reduce mineral loss. Despite their effectiveness, efforts continue to optimize prevention and to explore adjunctive technologies that might further increase enamel resistance to acid challenges.

Fluoride varnishes are high-concentration topical agents widely used as a non-invasive intervention to prevent enamel demineralization, manage incipient caries, and treat dentin hypersensitivity [1, 2]. On contact with saliva they harden into an adherent layer that sustains fluoride release, promoting the precipitation of a fluoride reservoir and the substitution of hydroxyl ions within hydroxyapatite to form the less soluble fluorapatite [3]. Systematic reviews have concluded that professionally applied 5% sodium fluoride varnish supports remineralization of incipient enamel lesions [4, 5].

Lasers have also been investigated for caries prevention because of their ability to modify enamel structure. Laser irradiation is thought to reduce tissue permeability and to alter the mineral phase, decreasing carbonate content while favouring a more acid-stable hydroxyapatite, thereby limiting acid diffusion during a cariogenic challenge [6]. Evidence remains scarce and inconsistent, however [7–12]. Over the last decade the Nd: YAG laser has attracted particular attention for improving acid resistance and reducing enamel permeability [8–10]. A clinical trial reported that combined Nd: YAG and topical fluoride reduced caries incidence in vivo [11], and other studies found reduced tooth dissolution [12] and morphologically hardened enamel when the laser was combined with fluoride agents [13]. Conversely, an in vitro study in primary teeth found that Nd: YAG combined with fluoride was no more effective than fluoride alone [14]. Reports on sequencing are contradictory, as one found improved performance when the laser preceded varnish [15], while another reported that irradiation before acidulated phosphate fluoride (APF) gel accelerated lesion progression [7]. Zezell et al. compared Nd: YAG and Er, Cr: YSGG irradiation and reported a significant reduction in enamel demineralization when APF gel was applied prior to laser treatment, together with compositional and crystallographic changes in the irradiated surface [16].

Several in vitro methods have been used to evaluate demineralization after laser irradiation, including scanning microradiography [17], surface microhardness [18], and micro-CT [19]. Micro-CT is a well-established, non-destructive technique that reconstructs the three-dimensional distribution of X-ray attenuation, which depends on photon energy and the atomic composition of the tissue [19]; it therefore allows both surface loss and subsurface radiodensity to be assessed on the same specimen.

This study evaluated the interaction between Nd: YAG laser irradiation and fluoride varnish in preventing bovine enamel demineralization, with laser power and treatment sequence as variables. Two null hypotheses were tested: (i) laser power (0.5, 0.75, 1.0 W) would not affect enamel resistance to the cariogenic challenge; and (ii) the order of laser and fluoride application would not affect enamel resistance.

## Materials and methods

### Study design and specimen preparation

Forty sound bovine incisors were disinfected in 2% chloramine for 10 days. From their buccal surfaces, 110 enamel blocks (5 × 4 × 5 mm) were obtained and randomly allocated to five groups: Control (C), Fluoride (F), Laser (L), Fluoride-then-Laser (FL), and Laser-then-Fluoride (LF). In the irradiated groups (L, FL, LF), each of the three power subgroups (0.5, 0.75, 1.0 W) contained 10 blocks. Because more than one block was obtained from some teeth, tooth of origin was recorded for each block.

Blocks were prepared with a low-speed handpiece (KaVo, Biberach, Germany) and a flexible diamond disc (KG Sorensen, São Paulo, SP, Brazil), embedded in self-curing acrylic resin with the enamel exposed, and wet-polished (Lapping and Polishing Machine, South Bay Technology, San Clemente, CA, USA) under water cooling with silicon-carbide papers of 600, 800, and 1200 grit (3 M, São Paulo, SP, Brazil) for 60 s each, followed by 1 μm diamond suspension (MetaDi II, Buehler, Lake Bluff, IL, USA) on a felt disc for 120 s. Specimens were ultrasonicated (USC-1400, Unique, Indaiatuba, SP, Brazil) for 20 min and stored humid until analysis. To delimit the test field, part of each surface was coated with cosmetic nail varnish (Colorama, São Paulo, SP, Brazil) and utility wax, leaving an exposed area of approximately 10 mm² for treatment; the coated remainder served as the internal reference area.

### Fluoride treatment protocol

A single thin layer of fluoride varnish (Duraphat, 5% NaF; Colgate-Palmolive, São Paulo, SP, Brazil) was applied with a microbrush (KG Sorensen, São Paulo, SP, Brazil). Specimens were kept humid for 4 h, after which the varnish was removed with a nº 15 scalpel blade.

### Nd: YAG laser irradiation protocol

Irradiation was performed using an Nd: YAG laser (Fidelis Plus III, Fotona, Ljubljana, Slovenia), applied perpendicular to the enamel surface with a 300 μm optical fiber in scanning mode at approximately 1 mm distance. Each specimen received two consecutive irradiations of 15 s. Before irradiation, a paste of charcoal powder in deionized water and 99% ethanol was applied as an exogenous chromophore to enhance surface absorption and localize heating to the superficial enamel.

Laser parameters varied according to subgroups (0.5, 0.75, and 1 W), with a frequency of 10 Hz and pulse duration of 300 µs, corresponding to energy densities of 150, 225, and 300 J/cm².

### Group allocation

The distribution of specimens according to treatment protocols is presented in Table 1.

**Table 1:** Treatment protocols

Groups	Subgroups	Surface treatment protocols
1. Control (C)	-	None
2. Fluoride (F)	-	Fluoride varnish
3. Nd: YAG laser (L)	L0.5 W / L0.75 W / L1W	Nd: YAG Irradiation
4. Fluoride /Laser (FL)	FL0.5 W / FL0.75 W / FL1W	Fluoride varnish, then Nd: YAG irradiation
5. Laser/Fluoride (LF)	LF0.5 W / LF0.75 W / LF1W	Nd: YAG irradiation, then fluoride varnish

### Demineralization protocol

Demineralization followed Wu and Fried [20]. Specimens were immersed in a solution of 2 mmol/L calcium, 2 mmol/L phosphate, and 0.075 mol/L acetate buffer (pH 4.5, adjusted with TRIS) at 37 °C for 48 h, with the solution renewed at 24 h. Coatings were then removed, specimens rinsed with distilled water, and stored humid until analysis. A continuous static challenge was used.

### Micro-computed tomography

Specimens were scanned in a microfocus X-ray computed tomography system (XT H 225 ST, Nikon Metrology, Tring, Hertfordshire, United Kingdom). Scanning was performed at 80 kV and 222 µA, without filter, with a pixel size of 11 μm and acquisition time of 25 min per specimen, generating 3017 axial projections. Images were reconstructed using XT-CT Pro 3D software (Nikon Metrology, Tring, Hertfordshire, United Kingdom). A volume of interest (VOI) corresponding to the enamel fragment embedded in acrylic resin was selected from the reconstructed dataset. The selected volumes were imported into VGStudio MAX 2.2 (Volume Graphics GmbH, Heidelberg, Germany), where a 3 × 3 × 3 Gaussian filter was applied to reduce noise and imaging artifacts. Grayscale values were linearly calibrated against air and water as reference points, with air set to 0 and water to 1000, and are reported as calibrated grayscale units (GSU).

Image processing was performed using ImageJ software, version 1.49u (National Institutes of Health, Bethesda, MD, USA). Regions of interest (ROI) measuring 100 × 100 pixels were defined in exposed (EA) and non-exposed areas (NEA). Each ROI comprised approximately 300 axial slices, with a voxel size of 11 μm. Mineral loss between EA and NEA, as well as enamel mineral density, were determined based on the grayscale values within the selected ROIs.

### Outcome measurement

#### Enamel

**Fig. 1:**
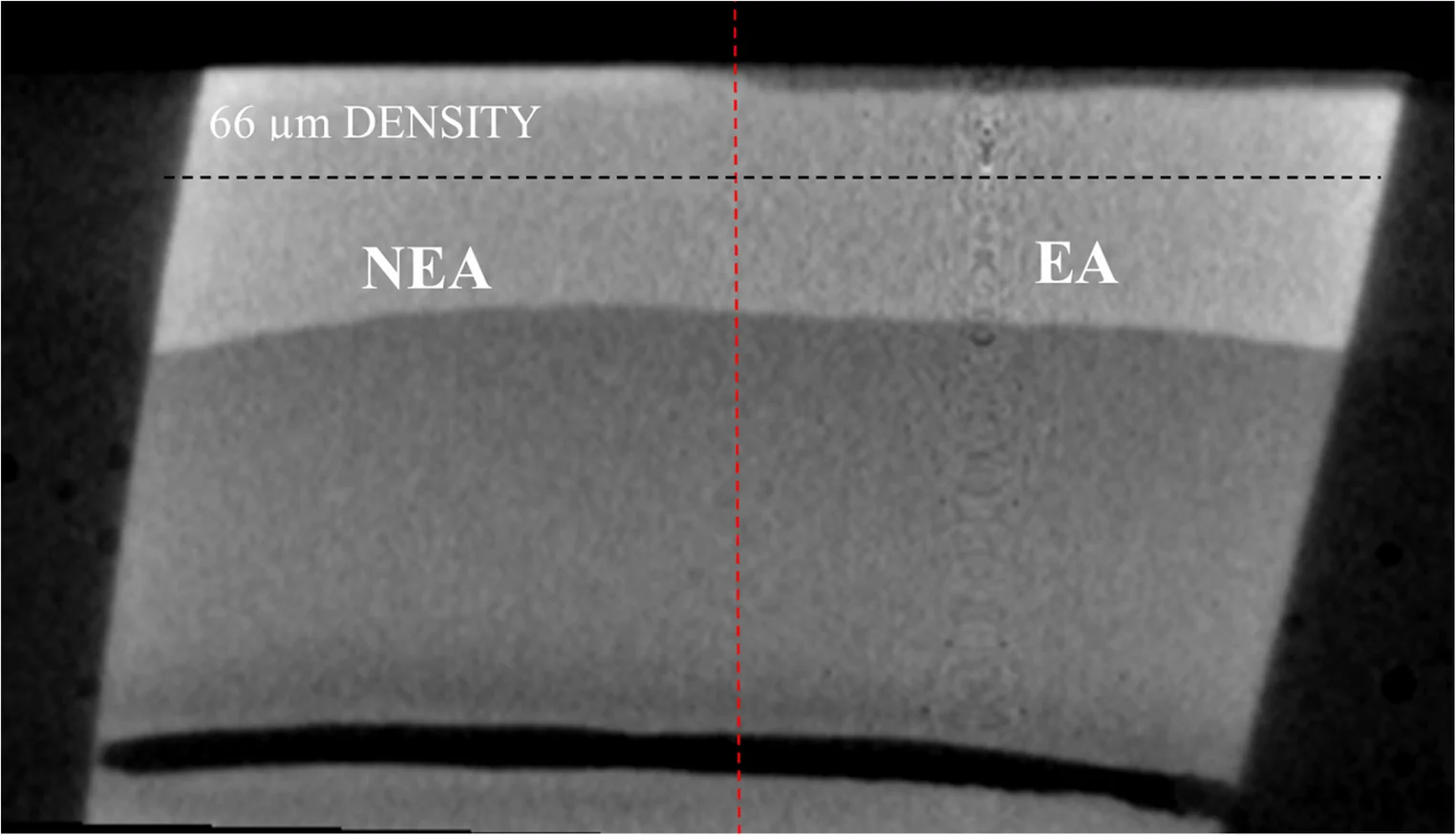
Coronal section of a control group specimen showing the exposed (EA) and non-exposed areas (NEA), separated by a red dashed line. The analyzed depth for radiodensity (up to 66 μm) is indicated by a black dashed line. Surface loss was determined as the difference between the NEA and EA.

Using ImageJ, regions of interest of 100 x 100 pixels spanning approximately 300 axial slices (11 µm voxel) were defined in the EA and NEA areas. Surface loss was measured as the vertical step-height between the outer boundary of the NEA and the receded surface of the EA (Figure 1) and expressed in micrometres. This is a geometric measure of surface recession, distinct from integrated mineral loss (ΔZ). 

#### Relative radiodensity difference (GSU)

**Fig. 2:**
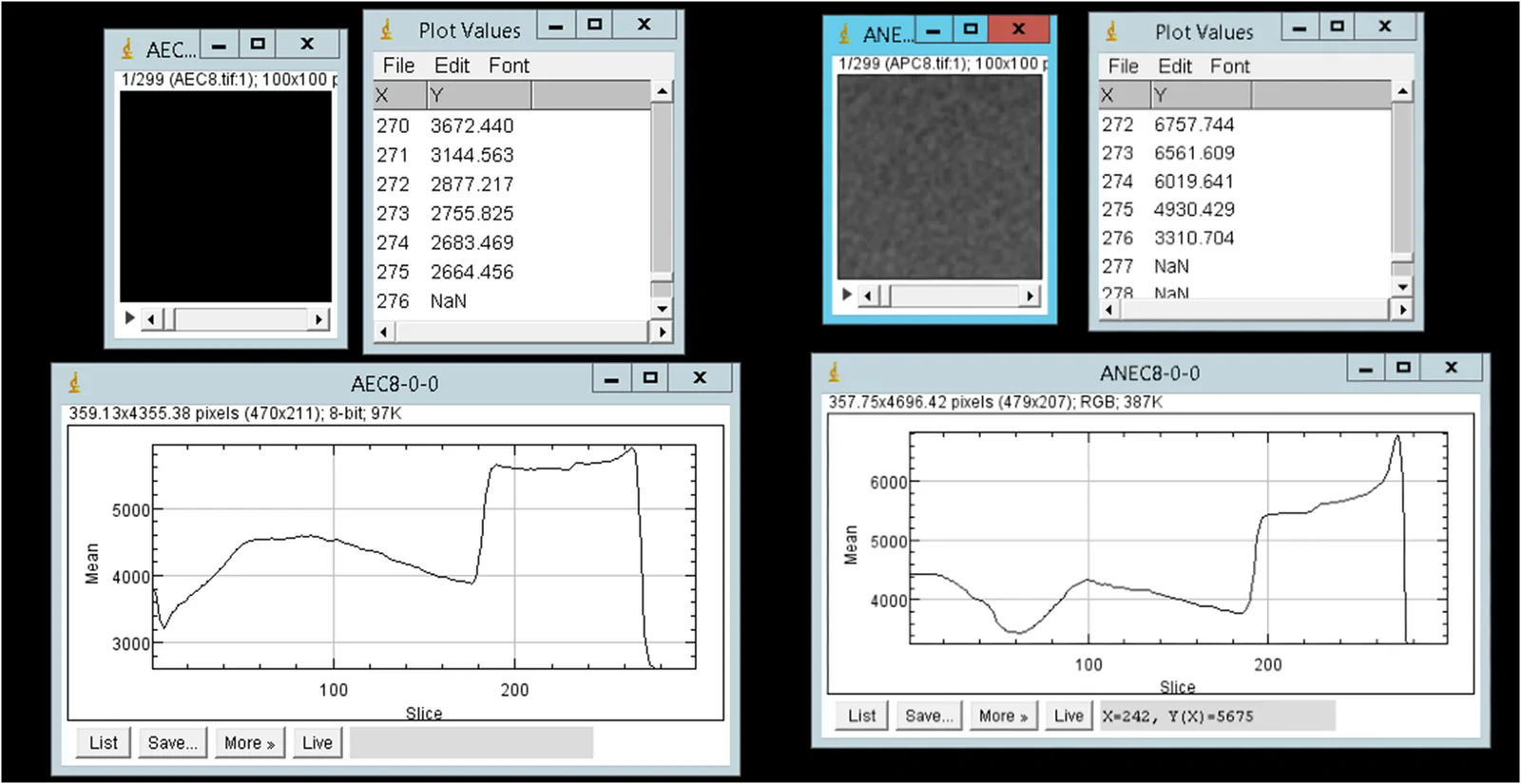
Determination of relative radiodensity by depth profiling. Regions of interest in the exposed (a) and protected (d) areas of a control specimen, with the corresponding slice-by-slice grayscale values (b, e) and their graphical distribution as a function of depth (c, f). Grayscale was sampled from the outer surface towards the interior of the specimen; the step visible in each curve corresponds to the enamel–dentine transition. Analysis was restricted to the six voxels nearest the surface, equivalent to a depth of 66 μm.

Using the Plot Z-axis Profile tool in ImageJ, the mean grayscale value was sampled over six sequential voxels (66 µm depth) from the surface inward in the protected and exposed areas, and the PA-EA difference was taken as a proxy for subsurface mineral change. The resulting profiles record grayscale as a function of depth from the outer surface towards the interior of the specimen. The transition between enamel and dentine is identifiable as a step in the curve, and sampling was restricted to the outermost six voxels, well above this transition (Figure 2).

#### Percentage protection

For both outcomes, the protective effect of each surface treatment was expressed as the percentage reduction in the outcome value relative to the untreated control group, calculated as:

$$\begin{array}{c}Protection\;\left(\%\right)\\=\;\left[1-\left(mean\;of\;treatment\;group\div mean\;of\;control\;group\right)\right]\times\;100\\\end{array}$$

The calculation was applied separately to surface loss and to the relative radiodensity difference. Values approaching 100% indicate that the treated area behaved similarly to the protected reference area, whereas values approaching zero indicate behaviour similar to untreated enamel. Percentage protection is a descriptive summary derived from group means and was not subjected to inferential testing.

### Statistical analysis

Data were tested for normality using the Kolmogorov-Smirnov test. As the data were normally distributed, one-way ANOVA was applied, followed by Tukey’s post hoc test for multiple comparisons. Results are expressed as mean ± standard deviation, with significance set at *p* < 0.05. All analyses were performed in SPSS version 20.0 (IBM Corp., Armonk, NY, USA).

## Results

The untreated control showed the greatest surface loss, with a mean step-height of 89.8 ± 24.6 μm between exposed and protected enamel (Table 2). One-way ANOVA detected differences among groups (*p* < 0.001). In the Tukey post hoc test, all treatments except FL0.5 significantly reduced surface loss relative to control (*p* < 0.05).

**Table 2:** Mean surface loss (lesion step-height, µm ± SD) by group (n = 10 per subgroup)

	Treatment	Mean surface loss (μm)	Protection (%)
1	C	89.8 ± 24.6ᵃ	-
2	F	33.8 ± 23.9ᵇᶜ	
3	L0.5	56.7 ± 19.0ᵇ	36.9
	L0.75	49.1 ± 25.0ᵇ	45.3
	L1	41.6 ± 19.4ᵇᶜ	53.7
4	FL0.5	68.8 ± 15.9ᵃ	23.5
	FL0.75	50.1 ± 17.9ᵇ	44.2
	FL1	36.4 ± 14.4ᵇᶜ	59.5
5	LF0.5	15.1 ± 11.0ᶜ	83.2
	LF0.75	14.5 ± 10.9ᶜ	83.9
	LF1	11.2 ± 12.4ᶜ	87.5
	*p*-value	< 0.001	62.4

One-way ANOVA, p < 0.001. Values sharing a superscript letter do not differ significantly (Tukey, p ≥ 0.05)

The Laser-then-Fluoride (LF) sequence produced the lowest surface loss at every power, ranging from 11.2 ± 12.4 μm at 1.0 W to 15.1 ± 11.0 μm at 0.5 W. The LF subgroups did not differ significantly from the Fluoride (33.8 ± 23.9 μm), L1 (41.6 ± 19.4 μm) or FL1 (36.4 ± 14.4 μm) groups (*p* ≥ 0.05), and differed significantly from FL0.5 (68.8 ± 15.9 μm). Surface loss did not differ significantly among L0.5, L0.75 and L1. Percentage protection against surface loss relative to control ranged from 23.5% in FL0.5 to 87.5% in LF1 (Table 2).

Subsurface radiodensity differences between EA and NEA were greatest in the control group (1756 ± 746 GSU; *p* < 0.001) and smallest in the Fluoride and Laser-then-Fluoride groups (Table 3). The lowest values were 317 ± 401 GSU in LF1 and 376 ± 292 GSU in F, which did not differ significantly from each other (*p* ≥ 0.05). Percentage protection of subsurface radiodensity ranged from 21.6% in L0.5 to 81.9% in LF1 (Table 3).

**Table 3:** Mean difference in relative radiodensity (calibrated grayscale units, GSU ± SD) between exposed and protected areas by group

	Treatment	Mean difference in radiodensity (GSU) + SD	Protection (%)
1	C	1756 ± 746ᵃ	-
2	F	376 ± 292ᶜ	
3	L0.5	1376 ± 590ᵃᵇ	21.6
	L0.75	1087 ± 624ᵃᵇ	38.1
	L1	1276 ± 614ᵃᵇ	27.3
4	FL0.5	1078 ± 815ᵃᵇ	38.6
	FL0.75	746 ± 574ᵇᶜ	57.5
	FL1	1299 ± 528ᵃᵇ	26
5	LF0.5	474 ± 531ᵇᶜ	73
	LF0.75	598 ± 333ᵇᶜ	65.9
	LF1	317 ± 401ᶜ	81.9
	*p*-value	< 0.001	78.6

One-way ANOVA, p < 0.001. Values sharing a superscript letter do not differ significantly (Tukey, p ≥ 0.05)

**Fig. 3:**
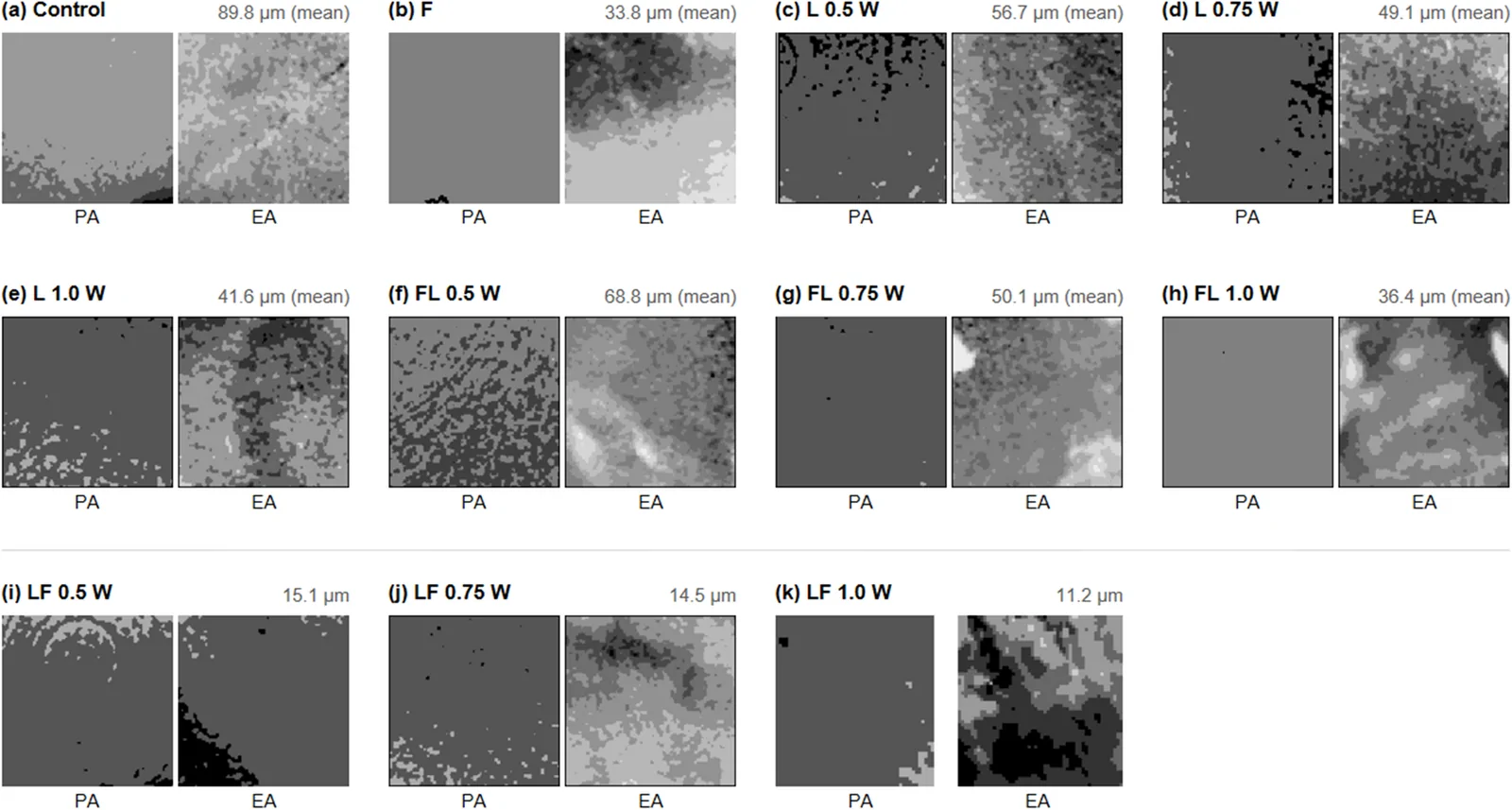
Micro-CT surface maps of the protected (PA) and exposed (EA) areas of enamel. Panels (a-k) show, for each group, the specimen whose surface loss value lay closest to the group mean; the value indicated is the group mean (Table 2). Each panel corresponds to a region of interest of 100 × 100 voxels (1.1 × 1.1 mm; 11 μm voxel), except (k), derived from a 60 × 60 voxel region. Grayscale represents relative height and was scaled individually for each image; panels are not quantitatively comparable between groups. Panels correspond to the control, fluoride, laser, fluoride-then-laser and laser-then-fluoride groups; quantitative data for all groups are reported in Tables 2 and 3

Representative micro-CT surface maps of the protected and exposed areas of each group are presented in Fig. 3.

## Discussion

In this in vitro study comparing Nd: YAG laser irradiation and fluoride varnish for the prevention of enamel demineralization, the sequence of application altered the outcome whereas the laser power did not. Irradiation performed before varnish application produced the lowest surface loss at every power tested, from 11.2 to 15.1 μm against 89.8 μm in untreated enamel, with protection exceeding 80% throughout. This sequence did not, however, differ significantly from fluoride varnish applied alone, which yielded 33.8 μm of surface loss. The reverse sequence behaved quite differently. Fluoride applied before irradiation conferred no measurable protection at 0.5 W, performing no better than untreated enamel.

Our findings align with the existing literature on combined laser and fluoride protocols while also elucidating why that literature has been inconsistent. Azevedo et al. [14] irradiated primary enamel before fluoride application at 0.5 W and found no enhancement beyond the laser alone, concordant with our observation that the combination, although superior to no treatment, was equivalent to fluoride monotherapy. Rios et al. [21] likewise reported a greater reduction of enamel wear when irradiation at 0.75 W preceded acidulated phosphate fluoride, although that advantage diminished over time. The recurrence of the sequence effect across models that differ in substrate, fluoride vehicle, and challenge supports the biological plausibility of a mechanism in which laser-induced surface modification precedes and facilitates fluoride uptake.

Previous investigations into laser power settings have demonstrated that Nd: YAG preserves enamel surface integrity and reduces demineralization at average powers between 0.6 and 1 W, while 1.2 W produces an ablative effect [7, 12, 22]. The present study evaluated 0.5, 0.75, and 1 W, which provided surface protection of 36.9%, 45.3%, and 53.7%, respectively (Table 2). Although protection increased numerically with power, the differences were not statistically significant, and a dose-response relationship cannot be inferred from these data. Any protective effect within this range may relate to reduced permeability, altered chemical composition, or modification of surface morphology, but the boundary between an effective and a damaging fluence remains to be defined.

The behaviour of the fluoride-first groups is mechanistically instructive, and two complementary processes may be involved. The first is attenuation of the incident beam. At 0.5 W these specimens were indistinguishable from untreated enamel, and protection improved only as power rose to 0.75 and 1.0 W. The most plausible explanation is that the overlying varnish and carbon layers attenuated the incident beam, so that insufficient energy reached the enamel to produce the protective structural change. This is consistent with the established thermal behaviour of enamel, in which carbonate is lost and acid phosphate condenses to pyrophosphate between roughly 100 and 650 °C, recrystallisation and crystal growth occur between 650 and 1100 °C, and surface melting narrows interprismatic spaces and impedes acid diffusion [8, 22–26]. Because Nd: YAG radiation is poorly absorbed by enamel, an exogenous chromophore was required to confine this heating to the superficial layer [23]. 

A second consideration concerns the interposed layers themselves. The varnish film and charcoal chromophore lie between the beam and the enamel, absorbing part of the incident energy. The dose-response observed among the FL subgroups supports this interpretation. Surface loss decreased as power increased, from 68.8 μm at 0.5 W to 50.1 μm at 0.75 W and 36.4 μm at 1.0 W, which aligns with the expectation that progressively more energy is required to compensate for attenuation. The subsurface data suggest the same. The FL groups retained radiodensity differences of 1078, 746, and 1299 GSU, which are on the same order as laser alone (1376, 1087, and 1276 GSU) and far from the 376 GSU recorded for fluoride alone. This suggests that whatever protection this sequence affords derives from laser-induced modification of the mineral phase rather than from an intact surface fluoride reservoir. The mechanism by which the interposed varnish limits this interaction was not investigated and remains a hypothesis.

Other investigations have applied micro-CT to quantify the effect of fluoride vehicles on enamel lesions. Shahmoradi et al. [27] imaged demineralized enamel by high-resolution micro-CT and extracted lesion depth and mineral density from density profiles, reporting a mean lesion depth of 86 ± 7.19 μm in untreated specimens against 67 ± 7.03 μm in varnish-treated specimens (*p* < 0.05), with mineral density in untreated lesions 25% lower than in varnish-treated enamel. The magnitude of the untreated lesion in that study is close to the 89.8 ± 24.6 μm recorded here for the control group, although the relative gain conferred by varnish alone was larger in the present model, which may reflect differences in the cariogenic challenge and in varnish contact time. Ahn et al. [28] followed enamel surface density by micro-CT at three depths in bovine incisors treated with fluoride varnish and reported that density decreased continuously in both the control and the varnish groups, that demineralized enamel volume was greatest in the control group followed by the varnish group, and that surface morphology was roughest and most irregular in the control group. This pattern is consistent with our findings, in which the control group showed both the greatest surface recession and the largest radiodensity difference between treated and reference areas, and supports the suitability of micro-CT for quantifying mineral change in enamel under laboratory challenge.

Not all evidence supports a favourable sequence effect. Dias-Moraes et al. [7] found that irradiation before acidulated phosphate fluoride gel failed to improve, and in their model accelerated, lesion progression, which they attributed to the formation of a more soluble calcium-fluoride-like layer. Differences in fluoride vehicle, in the nature and duration of the cariogenic challenge, and in the monitoring method may account for this divergence. The contrast underscores how sensitive these protocols are to experimental conditions and cautions against generalising a sequence effect observed under one set of conditions to another.

The clinical viability of the protocol warrants consideration. Nd: YAG irradiation of dental hard tissue depends on an exogenous chromophore, since the wavelength is poorly absorbed by enamel. The charcoal paste used here is a laboratory preparation rather than a product cleared for intraoral use. A clinical counterpart would need to be acceptable in taste, non-staining, safe if partially ingested, and straightforward to remove before varnish application. The irradiation protocol itself delivered 30 s, in two passes of 15 s, over an exposed area of approximately 10 mm². Extrapolated to the occlusal surface of a permanent molar, this corresponds to roughly two minutes of irradiation per tooth, to which chromophore application, its removal, and varnish application must be added. Full-mouth application would therefore be impractical, and the protocol appears better suited to selective use on surfaces at high risk, such as newly erupted molars or the teeth of patients with salivary hypofunction. Operational requirements also include adequate moisture control, protective eyewear for the patient and operator, plume management, and parameters that keep the intrapulpal temperature rise within safe limits. It should further be noted that the four-hour varnish contact adopted here was chosen to standardize the in vitro model and does not correspond to clinical practice, in which varnish is left in situ and removed by natural means.

Several limitations should be acknowledged. Surface temperature during irradiation was not monitored. Because the proposed protective mechanism is thermal, this limits mechanistic interpretation, and the safety margin of each power setting cannot be established from these data. Bovine enamel, while widely accepted as a substitute for human tissue, does not reproduce the complexity of the oral environment, and the static 48-hour challenge does not reproduce the cyclic demineralization and remineralization that occurs clinically. Radiodensity was expressed in grayscale units calibrated against air and water rather than hydroxyapatite phantoms, and therefore serves as a relative proxy rather than an absolute measure of mineral density. Because some enamel blocks were obtained from the same incisor, full specimen independence cannot be assumed. Finally, the analysis did not formally test the interaction between application sequence and laser power, and the subgroup comparisons should therefore be regarded as exploratory.

Future investigations should aim to validate these findings. Mechanistic studies exploring fluoride uptake, retention, and distribution following laser pretreatment would be valuable to clarify the underlying interactions between laser-induced enamel modification and chemical remineralization processes. The optimization of laser parameters, such as energy density, pulse duration, and irradiation mode, also remains necessary to define safe and effective clinical protocols. Given the well-established effectiveness, low cost, and ease of application of fluoride varnish, any additional benefit provided by laser therapy must be weighed against the practical constraints discussed above. Accordingly, future research should incorporate economic analyses and randomized clinical trials to determine whether adjunctive laser use offers a meaningful advantage in specific patient populations, particularly those at high risk of caries.

## Conclusion

The results indicate that laser irradiation prior to fluoride application was more effective than the reverse sequence, producing the lowest surface loss and the smallest subsurface radiodensity difference, whereas laser power did not influence the outcome. The anti-demineralization effect of the best laser-assisted protocol was comparable to that of fluoride varnish applied alone under the conditions tested. As these findings derive from a laboratory model, they cannot be extrapolated to clinical performance.

## Data Availability

The data that support the findings of this study are available from the corresponding author upon reasonable request.
